# Expressing Redundancy among Linear-Epitope Sequence Data Based on Residue-Level Physicochemical Similarity in the Context of Antigenic Cross-Reaction

**DOI:** 10.1155/2016/1276594

**Published:** 2016-05-04

**Authors:** Salvador Eugenio C. Caoili

**Affiliations:** Department of Biochemistry and Molecular Biology, College of Medicine, University of the Philippines Manila, Room 101, Medical Annex Building, 547 Pedro Gil Street, Ermita, 1000 Manila, Philippines

## Abstract

Epitope-based design of vaccines, immunotherapeutics, and immunodiagnostics is complicated by structural changes that radically alter immunological outcomes. This is obscured by expressing redundancy among linear-epitope data as fractional sequence-alignment identity, which fails to account for potentially drastic loss of binding affinity due to single-residue substitutions even where these might be considered conservative in the context of classical sequence analysis. From the perspective of immune function based on molecular recognition of epitopes, functional redundancy of epitope data (FRED) thus may be defined in a biologically more meaningful way based on residue-level physicochemical similarity in the context of antigenic cross-reaction, with functional similarity between epitopes expressed as the Shannon information entropy for differential epitope binding. Such similarity may be estimated in terms of structural differences between an immunogen epitope and an antigen epitope with reference to an idealized binding site of high complementarity to the immunogen epitope, by analogy between protein folding and ligand-receptor binding; but this underestimates potential for cross-reactivity, suggesting that epitope-binding site complementarity is typically suboptimal as regards immunologic specificity. The apparently suboptimal complementarity may reflect a tradeoff to attain optimal immune function that favors generation of immune-system components each having potential for cross-reactivity with a variety of epitopes.

## 1. Introduction

Immunological targeting of antigens exemplified by pathogen virulence factors, allergens, and even typical drugs is fundamental to the solution of global-health problems including both infectious and noninfectious diseases [1–3]. This entails molecular recognition of antigens by immune-system components (e.g., antibodies and T-cell receptors), which occurs via binding of epitopes (i.e., the recognized submolecular structural features of antigens) [4, 5]. Epitope prediction (i.e., computational identification of epitopes among biomolecules such as proteins) aims to enable selective incorporation of particular epitopes (e.g., actual targets of protective immune responses rather than disease-enhancing immunological decoys) into antigenic constructs (e.g., synthetic peptides) for novel vaccines, immunotherapeutics, and immunodiagnostics [6]. However, this is complicated by the limited accuracy of existing tools for epitope prediction [7]. The present work thus explores the crucial yet largely neglected issue of epitope-data redundancy as a key consideration in epitope-prediction tool development.

Progressive development of epitope-prediction tools requires empirical epitope data for both training (e.g., in the context of machine learning) and benchmarking [8–10]. Hence, epitope-data redundancy is a major concern especially where data-driven statistical and machine-learning methods are employed to develop tools for epitope prediction and also related applications (e.g., MHC binding prediction), as studies might yield biased results due to overrepresentation of similar epitopes. Similarity among epitopes is often expressed as sequence similarity, particularly for linear peptidic epitopes which include continuous B-cell epitopes (each consisting of a single unbroken epitope-residue sequence, in contrast to discontinuous epitopes wherein epitope residues are separated in the sequence by intervening residues) and typical T-cell epitopes (each bound by a MHC molecule for presentation to T-cells). For these, classical heuristic approaches to decrease the redundancy entail setting a similarity threshold (typically expressed as a fraction of identical residues for a pair of aligned sequences), such that subsequent analyses may compensate accordingly (e.g., by excluding sequences sharing a degree of similarity above the threshold). This practice is well-established for general-purpose protein structural analyses [11–13] but potentially problematic if applied to peptidic epitopes in view of the nonlinear relationship between sequence similarity and antigenic similarity (e.g., as demonstrated by radically divergent antigenic properties arising from a structural difference of only a single chemical group [14]). This suggests the need for a more functionally meaningful alternative approach to expressing redundancy of epitope data.

Protein folding and binding [15] may be regarded as manifestations of the same underlying phenomenon driven by the hydrophobic effect, favoring burial of nonpolar surfaces in general away from solvent water, albeit with more selective burial of polar surfaces that favors complementary pairing between hydrogen-bond donors and acceptors. Residues that thus become completely buried (e.g., within the core of a folded protein or at the binding interface of a ligand-receptor complex) are sterically and electrostatically constrained by surrounding residues, to a much greater extent than unfolded or even folded but only partially buried residues (e.g., at solvent-exposed protein surfaces). Consequently, molecular recognition of epitopes, which is mediated by local ligand-receptor binding interactions, depends on sequence details much more than overall (i.e., global) features of protein structure do, notably in the sense of protein folds.

Proteins may share the same fold (e.g., as demonstrated by structural superposition of their backbones) well into the so-called twilight zone below the threshold for reliable detection of aligned-sequence similarity (i.e., less than 35% pairwise sequence identity) [16, 17]. Residue substitutions can be tolerated at surface-exposed positions (e.g., with replacement of certain polar residues by others whose side-chains differ in steric and electrostatic properties). Even at buried-core positions, certain nonpolar residues may be replaced by others whose side-chains differ in volume, especially where additional substitutions or other changes compensate for the volume differences, although the introduction of unsatisfied hydrogen-bond donors or acceptors and of unpaired formal charges tends to be poorly tolerated [18, 19]. However, even just a single-residue substitution in an epitope may abolish epitope-specific immune binding (e.g., by antibodies) if surface complementarity is disrupted at the epitope-binding interface (much as the structural organization of a protein core is disrupted by a radical substitution), as observed with immune evasion by pathogen escape mutants. Hence, the notion of conservative residue substitutions as conceptualized for evolving protein sequences (e.g., on the basis of changes in both volume and hydrophobicity) is of questionable applicability to epitopes as it obscures crucial details of changes in steric and electrostatic complementarity.

From the perspective of immune function, redundancy of epitope data arguably implies potential for antigenic cross-reaction (i.e., recognition of different epitopes by a common B- or T-cell receptor or equivalent thereof) rather than sequence similarity defined without reference to any immune-system component. Hence, the present work outlines a generic theoretical framework for quantitatively expressing epitope data redundancy in terms of potential antigenic cross-reactivity as estimated from an idealized limiting case of optimal ligand-receptor complementarity (comparable to the steric and electrostatic self-complementarity observed within folded proteins [20]). This is applied to linear peptidic epitopes, for clarity of illustration and considering their envisioned roles as immune targets in relation to peptide-based vaccines and immunodiagnostics. Theoretical results are compared with informative available experimental data on antigenic cross-reactivity of structurally related short peptides, in light of possible suboptimal ligand-receptor complementarity and its biomedical implications vis-a-vis epitope sequence variation (e.g., arising as pathogen mutations and host polymorphisms).

## 2. Theory and Methods

### 2.1. Functional Redundancy of Epitope Data

The present work aims to support the use of available empirical data to further develop epitope-prediction methods, without discarding unique epitopes deemed redundant on the basis of some sequence similarity threshold. Epitope data thus retained must be characterized as to redundancy, for which a reduced epitope count is introduced below. This is based on epitope similarity defined in terms of either aligned-sequence similarity or functional similarity as regards cross-reactive epitope binding. Aligned-sequence similarity is explored first because it is a familiar and readily calculated quantity. However, its limitations become apparent in the context of attempting to describe antigenic cross-reaction from a physicochemical perspective. Hence, functional similarity is also explored.

For a set of epitopes (construed as epitope structures, e.g., linear peptidic sequences), functional redundancy can be expressed as a reduced epitope count *r* such that 1 ≤ *r* ≤ *n*, where *n* is the total epitope count, with *r* = 1 and *r* = *n*, respectively, corresponding to extreme cases wherein every epitope is either maximally or minimally similar to every other epitope from a functional standpoint. Thus, structurally identical epitopes would be maximally similar to one another; but structurally nonidentical epitopes might also be regarded as maximally similar if their structural difference was negligible or otherwise irrelevant from a functional standpoint. For simplicity, further elaboration of concepts herein focuses on cases wherein all epitopes are structurally unique peptidic sequences of equal length. (The qualifier of  “structurally unique” is consistent with real-world epitope databases insofar as they regard each epitope as a structurally unique entity. The qualifier of “equal length” is clearly applicable to typical class I MHC-restricted T-cell epitopes and relatively short linear B-cell epitopes, albeit less so for longer B-cell epitopes and class II MHC-restricted T-cell epitopes.)

For a set of *n* structurally unique epitopes, the reduced epitope count may be defined as

(1) $$\begin{array}{c} r=\overset{n}{\underset{i=1}{\sum }}w_{i}, \end{array}$$

where *w*_*i*_ is the contribution of the *i*th epitope to *r*. In turn, *w*_*i*_ may be defined as

(2) $$\begin{array}{c} w_{i}=\frac{1+\sum_{j=1}^{n}\left(1-S_{ij}\right)}{n}, \end{array}$$

where *S*_*ij*_ is the functional similarity between the *i*th and *j*th epitopes, such that 0 ≤ *S*_*ij*_ ≤ 1, with *S*_*ij*_ = 0 and *S*_*ij*_ = 1, respectively, corresponding to extreme cases wherein the *i*th and *j*th epitopes are either minimally or maximally similar from a functional standpoint. If all the epitopes are of uniform sequence length *m* amino-acid residues, *S*_*ij*_ might be defined for a pairwise epitope sequence alignment as

(3) $$\begin{array}{c} S_{ij}=\frac{\sum_{k=1}^{m}s_{ijk}}{m}, \end{array}$$

where *s*_*ijk*_ is the functional similarity between amino-acid residues *a*_*ik*_ and *a*_*jk*_ at the *k*th sequence positions of the aligned *i*th and *j*th epitope sequences, respectively, such that 0 ≤ *s*_*ijk*_ ≤ 1, with *s*_*ijk*_ = 0 and *s*_*ijk*_ = 1, respectively, corresponding to extreme cases wherein *a*_*ik*_ and *a*_*jk*_ are either minimally or maximally similar from a functional standpoint. Accordingly, *s*_*ijk*_ might be simply defined as

(4) $$\begin{array}{c} s_{ijk}=\left\{\begin{array}{cc} 0, & \text{if }a_{ik}\neq a_{jk}\\ 1, & \text{if }a_{ik}=a_{jk}, \end{array}\right. \end{array}$$

for (3) to yield the fraction of identical aligned residues, which is a conventional measure of protein sequence similarity [11–13]. However, this is an oversimplification that fails to capture the possibility of minimal functional similarity between epitopes (e.g., operationally defined as undetectable antigenic cross-reactivity in an immunoassay) arising from a structural difference of only a single chemical group [14], unless the possibility of antigenic cross-reaction is denied altogether.

A more realistic physicochemically grounded alternative is to conceptualize functional similarity on the basis of biomolecular binding interactions, thereby relating functional similarity to binding affinity as measured via an immunoassay that employs some epitope-binding probe (e.g., antibody). Thus, functional similarity between epitopes (i.e., *S*_*ij*_ of (2) and (3)) may be defined alternatively as the Shannon information entropy [34–36] for the equilibrium distribution of epitope-bound probe between the *i*th and *j*th epitopes, with both epitopes at the same concentration in the presence of much less probe, such that

(5) $$\begin{array}{c} S_{ij}=-\left(\;f_{i}\log_{2}f_{i}+f_{j}\log_{2}f_{j}\;\right), \end{array}$$

where *f*_*i*_ and *f*_*j*_ are the fractions of epitope-bound probe bound to the *i*th and *j*th epitopes, respectively (noting that *f*_*i*_ + *f*_*j*_ = 1; and *S*_*ij*_ = 1, where the *i*th and *j*th epitopes are indistinguishable by means of the immunoassay). Constraining *f*_*j*_ as *f*_*j*_ ≤ *f*_*i*_, it may in turn be defined as

(6) $$\begin{array}{c} f_{j}=\frac{\prod_{k=1}^{m}s_{ijk}}{1+\prod_{k=1}^{m}s_{ijk}}, \end{array}$$

where *s*_*ijk*_ retains its meaning as introduced in (3). However, retaining the definition of *s*_*ijk*_ given by (4) would be tantamount to denying the possibility of antigenic cross-reaction; hence, an alternative is warranted.

A plausible starting point is the essential unity of protein folding and binding phenomena based on steric and electrostatic complementarity of molecular surfaces as observed within typical folded proteins [20]. If this is regarded as representative of ligand-receptor interfaces wherein the ligand is a peptidic epitope bound to one or more protein receptors (e.g., antibody, or MHC molecule, and T-cell receptor), an idealized epitope-binding site may be devised to estimate epitope similarity as potential antigenic cross-reactivity in terms of structural differences, assuming optimal complementarity with all epitope atoms completely surrounded by and close-packed against the binding-site contact atoms. Hence, suboptimal complementarity would arguably result if even a single epitope amino-acid residue was structurally altered. Notably, steric clashes conceivably would preclude antigenic cross-reaction if the altered residue was sterically incompatible with its original counterpart in the sense of failing to assume any conformation allowing it to fit entirely within the region of space (as defined by residue van der Waals surfaces) that otherwise would have been occupied by the original residue at the binding site. Thus, functional similarity between epitope amino-acid residues (i.e., *s*_*ijk*_ of (3), (4), and (6)) may be defined alternatively as

(7) sijk=0,if  aik,ajk  sterically  incompatibleexp⁡−ΔΔGijkRT,otherwise,

where *i* and *j* denote the original and altered epitopes, respectively; *a*_*ik*_ and *a*_*jk*_ retain their meanings as in (4); ΔΔ*G*_*ijk*_ is the contribution to the change in the overall free-energy change of epitope binding, due to replacement of *a*_*ik*_ by *a*_*jk*_; *R* is the gas constant; and *T* is the absolute temperature. ΔΔ*G*_*ijk*_ is analogous to the change in the free-energy change of folding due to replacement of the *k*th residue of a protein by a structurally different residue (e.g., to generate a mutant version of a wild-type protein).

**Figure 1:**
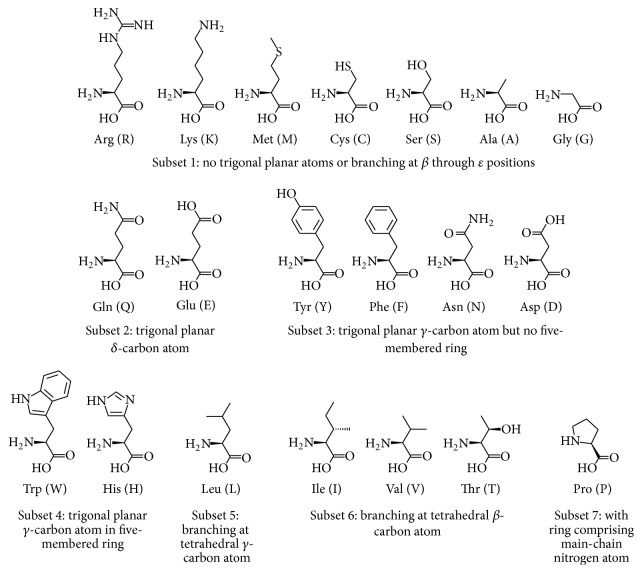
Subsets of the 20 proteinogenic amino acids grouped by stereochemical similarity. Structures were obtained from the ChEBI (Chemical Entities of Biological Interest) database (http://www.ebi.ac.uk/chebi/). Within each subset comprising two or more amino acids, these are arranged from left to right in order of decreasing size, such that sterically incompatible changes are avoided by substitution of a smaller amino acid for a larger one. The subsets are linked to form the residue substitution paths in Figure 2, wherein Subset 1 comprises an entire path while the other subsets comprise the upstream segments of paths converging on either Cys (C) or Ala (A).

To evaluate (7), steric incompatibility is posited for every substitution wherein the net change in volume is positive (i.e., wherever bulk increases) and also for other substitutions wherein stereochemical differences (e.g., relating to bond angles and branching) are deemed sufficient to result in steric clashes that preclude antigenic cross-reaction. Steric incompatibility is conceptualized herein on the basis of grouping the 20 proteinogenic amino acids into subsets according to stereochemical similarity, as depicted in Figure 1. Subset assignments are based on the covalent bonding geometry of nonhydrogen side-chain atoms. In particular, these atoms are characterized as either tetrahedral or trigonal planar, and branching of tetrahedral atoms is also considered. For example, methyl and methylene carbon atoms are tetrahedral, as are sulfur and amine nitrogen atoms. Hence, the sulfhydryl group of Cys is regarded as isosteric with a methyl group, whereas the sulfur atom of Met and the side-chain imino group of Arg are both regarded as isosteric with a methylene group. In contrast, amide and carboxyl carbon atoms are trigonal planar rather than tetrahedral.

**Figure 2:**
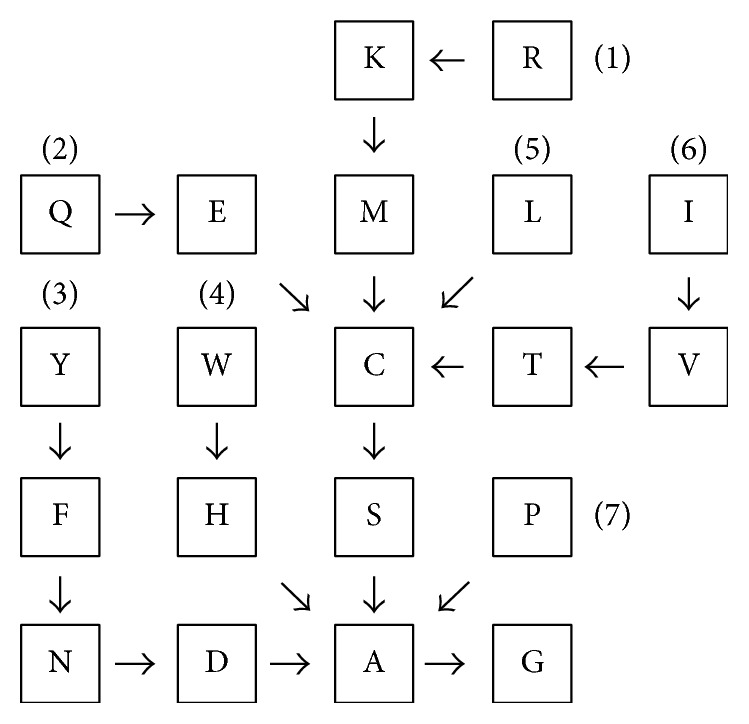
Amino-acid residue substitution paths avoiding sterically incompatible changes (cf. (7)) for the 20 canonical proteinogenic residues. Numbers correspond to the subsets of stereochemically similar amino acids depicted in Figure 1. For every residue substitution along the paths shown, the number of unsatisfied hydrogen bonds is presented in Table 1.

The subsets defined in Figure 1 are linked to form residue substitution paths avoiding sterically incompatible changes, as depicted in Figure 2. Subset 1 comprises an entire path while the other subsets comprise the upstream segments of paths converging on either Cys or Ala. The path of Subset 2 converges on Cys, as members of Subset 1 larger than Cys are sterically incompatible with Subset 2, whose *δ*-carbon atom is trigonal planar. The path of Subset 3 converges on Ala rather than Cys, because the *γ*-carbon atom is trigonal planar in Subset 3. The case of Subset 4 is thus similar to that of Subset 3, but the *γ*-carbon atom in Subset 4 is constrained as part of a five-membered ring, such that Subsets 3 and 4 are deemed sterically incompatible. The path of Subset 5 (Leu) converges on Cys, as members of Subset 1 larger than Cys are sterically incompatible with the branching at the *γ*-carbon atom of Leu. The path of Subset 6 converges on Ala instead of Cys, because of the branching at the *β*-carbon atom of Subset 6. Finally, the path of Subset 7 (Pro) converges on Ala, as the Pro side-chain is constrained by ring formation with the main-chain nitrogen atom and thus distorted beyond the *β*-carbon atom.

According to Figure 2, a substitution from any residue upstream to any residue downstream along a particular path avoids steric incompatibility. Conversely, downstream-to-upstream and off-path substitutions are deemed sterically incompatible. This is a qualitative first approximation using a highly idealized model, rather than an attempt at definitive categorization.

As regards ΔΔ*G*_*ijk*_ (in (7)), this is related to amino-acid residue structural differences (as a qualitative first approximation, rather than an attempt to obtain quantitatively very accurate results) by heuristic partitioning as

(8) $$\begin{array}{c} \Delta \Delta G_{ijk}=c\Delta V_{ijk}+bN_{ijk}, \end{array}$$

where *c* and *b* are both proportionality constants while Δ*V*_*ijk*_ is the change in residue volume and *N*_*ijk*_ is the number of unsatisfied hydrogen bonds (Table 1, wherein the residues are ordered by their volumes [21]), considering that an energetic penalty is incurred with unsatisfied hydrogen bonds upon binding a suboptimally complementary epitope.

Table 1 is sparse, with a null value (“—”) for most cells, because (8) is evaluated only for sterically compatible substitutions (cf. (7)). Hence, the numbers in Table 1 are for sterically compatible substitutions according to Figure 2. The said numbers were each obtained assuming that every available highly electronegative atom participates in the formation of a hydrogen bond, as

(9) $$\begin{array}{c} N_{ijk}=A_{ijk}-B_{ijk}, \end{array}$$

where *A*_*ijk*_ is the total number of oxygen and nitrogen atoms in the aligned residues while *B*_*ijk*_ is the number of such atoms for which direct correspondence was affirmed as regards structural superposability, atom identity, and covalent bonding partners (according to Figure 1). Thus, *N*_*ijk*_ increases with each failure to affirm a direct correspondence between hydrogen-bonding atoms. Physically, such failure corresponds to an unsatisfied hydrogen bond on the epitope, its binding site, or both.

Direct correspondence was affirmed for all main-chain (i.e., backbone) carbonyl oxygen atoms. In view of the difference between Pro and other residues at the main-chain nitrogen atom, direct correspondence was affirmed for this atom between Pro and itself as well as between non-Pro residues. Among side-chain atoms, direct correspondence was affirmed only between hydroxyl oxygen atoms of Ser and Thr, carbonyl oxygen atoms of Asp and Asn and of Glu and Gln, and *δ*-nitrogen atoms of His and Trp. To facilitate explication, a residue is termed polar if its side-chain contains at least one oxygen or nitrogen atom but nonpolar if otherwise. Accordingly, *N*_*ijk*_ = 0 for pairs of identical residues (along the diagonal of Table 1) and of non-Pro nonpolar residues. For Pro paired with Gly or Ala, *N*_*ijk*_ = 1 due to the main-chain nitrogen difference. For a polar residue paired with a non-Pro nonpolar residue, *N*_*ijk*_ is the total number of side-chain oxygen and nitrogen atoms. For a pair of polar residues, the total number of side-chain oxygen and nitrogen atoms is an upper bound on *N*_*ijk*_. Hence, *N*_*ijk*_ = 3 for Tyr paired with Asn, although *N*_*ijk*_ = 2 for Asn paired with Asp.

Provisional values for the proportionality constants *c* and *b* in (8) were obtained from literature. In particular, *c* was regarded as the energetic penalty per unit volume of a cavity formed due to substitution of a less bulky residue within the interior of a folded protein, with value of −0.024 kcal mol^−1^ Å^−1^ estimated empirically from protein mutation studies [37]; and *b* was likewise regarded as the energetic cost of breaking a hydrogen bond, with a conservative estimated value of 0.5 kcal mol^−1^ per bond [38].

Thus using (1) through (8) in conjunction with Table 1, the literature values for constants in (8), and published amino-acid residue volumes [21], reduced epitope counts (1) were computed for artificial epitope sequences and also actual experimentally studied epitope sequences, assuming a temperature of 37°C (310.15 K).

### 2.2. Retrieval and Processing of Epitope Data

**Figure 3:**
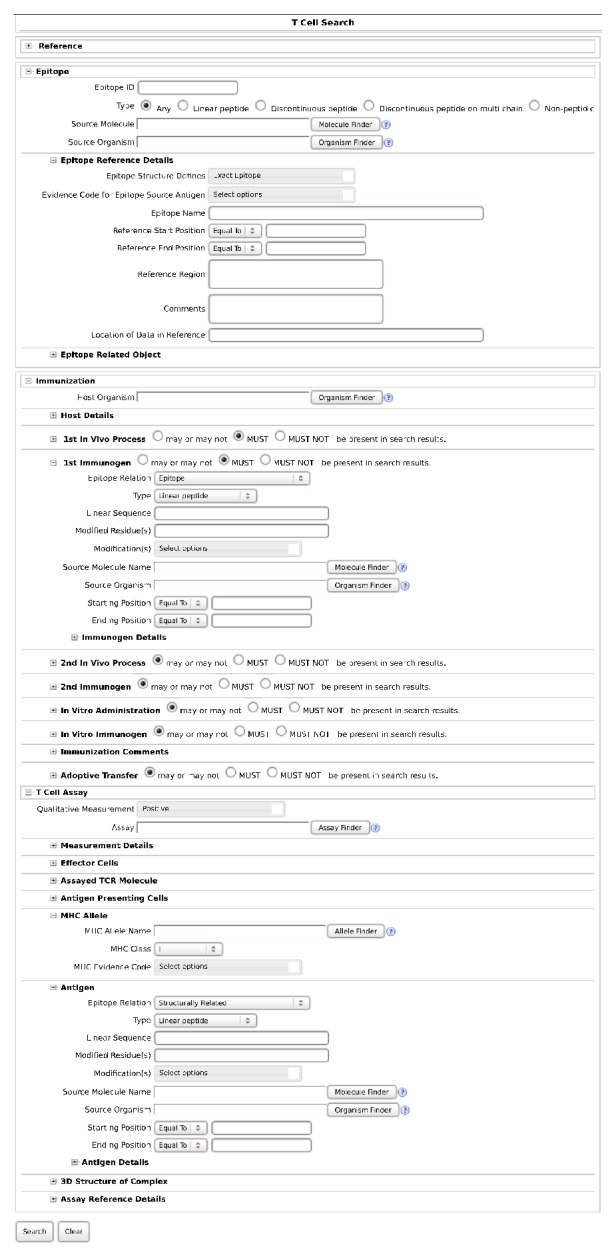
IEDB T-Cell Search facility interface (http://www.iedb.org/advancedQueryTcell.php). Example shown corresponds to narrow search for class I MHC-restricted T-cell assays with positive data on cross-reactivity between epitopes differing by only a single amino-acid residue substitution. Searches for B-cell assays were performed using IEDB B-Cell Search facility interface (http://www.iedb.org/advancedQueryBcell.php) of analogous form (e.g., without assay-related fields for “MHC allele”); narrow searches for negative data were performed without specified value for field labeled as “Epitope Structure Defines”; and broad searches were performed without specified value for both said field and that named “Qualitative Measurement” (see main text for additional details.).

Epitope data were obtained via the Immune Epitope Database (IEDB, at http://www.iedb.org/) [39] as database records retrieved through searches conducted from 4 to 6 October 2015 using its B-Cell Search and T-Cell Search facilities, in the latter case restricting searches to either MHC class I or II alleles, with each record thus retrieved pertaining to an individual B-cell or T-cell assay and containing data fields defined in relation to the key concepts of “Object Type” (i.e., molecule type in the context of chemical structure), “Epitope Relation” (i.e., molecule relationship to epitope), “1st Immunogen” (i.e., immunogen initially administered to elicit the immune response), and “Antigen” (i.e., antigen used in the B-cell or T-cell assay). Searches were restricted such that the data fields named “1st Immunogen Object Type” and “1st Immunogen Epitope Relation” had values of “Linear Peptide” and “Epitope”, respectively, with the “MHC class” option set to either “I” or “II” for T-Cell Search. Employing this basic search strategy, both narrow and broad searches were conducted, the former to retrieve data on cross-reactivity between epitopes differing by only a single amino-acid residue substitution and the latter to survey the entire repertoire of available data on linear peptidic epitopes (as illustrated by example in Figure 3).

Narrow searches were first attempted to retrieve records for which structural data on epitope binding were available, setting the “Yes” option for the Viewer Flag (under “3D Structure of Complex”). Subsequently, the searches were conducted to separately retrieve records curated as containing either negative or positive data on cross-reactivity and were restricted such that the data fields named “Antigen Object Type” and “Antigen Epitope Relation” had values of “Linear Peptide” and “Structurally Related”, respectively, with the data field named “Qualitative Measurement” having a value of either “negative” or “positive.” In the latter case, searches were further limited to class I MHC-restricted T-cell assays for which the data field named “Epitope Structure Defines” had a value of either “Exact Epitope” (rather than “epitope containing region/antigenic site”), as cross-reaction may occur with structural differences outside any relevant epitopes unless this is for a peptide confined within a class I MHC binding cleft. For each record thus retrieved, the immunogen and antigen sequences (i.e., values of the data fields named “1st Immunogen Object Primary Molecule Sequence” and “Antigen Object Primary Molecule Sequence,” resp.) were compared. The record was considered for further processing only if the said sequences were of equal length and differed at exactly one amino-acid residue position, and the record was ultimately retained only if a corresponding record with a “Qualitative Measurement” data-field value of “positive” was found sharing the same immunogen and immunoassay protocol except that the antigen was identical to the immunogen (thus confirming that the immunogen had elicited a relevant anti-peptide immune response in the first place). The epitope data thus obtained were analyzed in light of the preceding Section 2.1.

The broad searches were conducted to retrieve records on linear peptidic epitopes in general. Among the epitope records thus retrieved, those wherein the data field named “Modification” was nonempty (indicating amino-acid residue covalent modification) were excluded from further consideration, and the remainder were analyzed as regards functional redundancy according to the preceding Section 2.1.

## 3. Results and Discussion

### 3.1. Functional Redundancy versus Sequence

**Figure 4:**
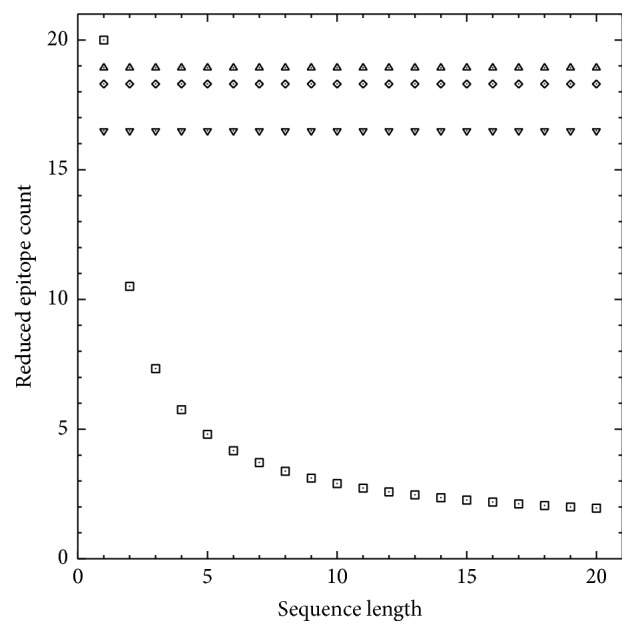
Reduced epitope counts (1) for peptidic sequences of uniform length varying only at a single-residue position. Each count corresponds to a set of 20 peptides representing every standard proteinogenic amino-acid residue at the variable-residue position. Functional similarity is equated with either fractional aligned-sequence identity (“□”; (3) and (4)) or the Shannon information entropy for differential epitope binding ((5) through (9)). In the latter case, counts were based on steric incompatibility only (“▽”; (7) and Figure 2), both steric incompatibility and cavity formation (“⋄”; *c*Δ*V*_*ijk*_ in (8)), or steric incompatibility with both cavity formation and hydrogen bonding (“△”; (8) and (9) and Table 1).

As depicted in Figure 4, computed functional redundancy increases with epitope sequence length if functional similarity is equated with fractional aligned-sequence identity (“□”), whereas it is independent of sequence length if functional similarity is equated with the Shannon information entropy for differential epitope binding (“▽,” “⋄,” and “△”). The latter approach thus better accounts for the possibility of a structural difference in only a single chemical group being sufficient to preclude antigenic cross-reaction provided that the sequence under consideration is entirely encompassed by the relevant epitope structure, as structural differences may be functionally irrelevant if they lie outside the epitope. Moreover, computed functional redundancy decreases (i.e., the reduced epitope count approaches the total epitope count) when both steric incompatibility and the energetic penalty of cavity formation are considered (“⋄”) instead of steric incompatibility alone (“▽”). The redundancy decreases even further if the energetic penalty of unsatisfied hydrogen bonds (more generally representing suboptimal electrostatic complementarity) is also considered (“△”).

### 3.2. Empirical Cross-Reactivity Data

**Figure 5:**
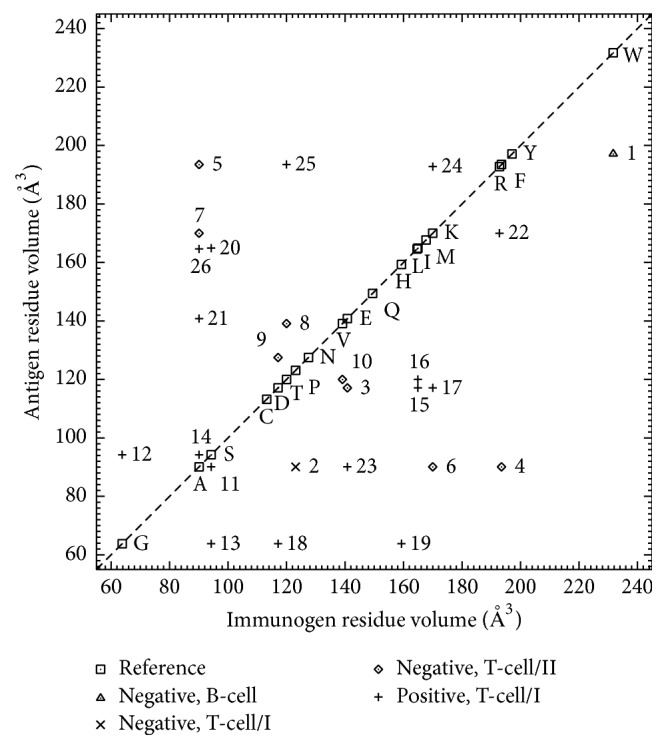
Peptide cross-reactivity assay data of Table 2 vis-a-vis amino-acid residue volume [21] of immunogen and antigen at single-residue substitution positions along sequence alignments.

IEDB searches yielded 26 records on epitope cross-reactivity vis-a-vis single-residue substitution (Table 2), all of which contain only qualitative rather than quantitative binding data, without reference to atomic coordinates or other structural details of bound epitopes. The data suggest that the posited steric incompatibility (in (7) and Figure 2) assumes too little tolerance for suboptimal complementarity, especially as regards volume differences between immunogen and antigen in Figure 5, which depicts examples of cross-reaction despite substitutions increasing residue bulk (i.e., positive-data points to the left of the diagonal). This might be at least partially corrected by relaxing or even eliminating the steric-incompatibility criteria, possibly replacing them with a continuous-form shape-dependent energetic penalty (considering that they assume an infinite-step hard-sphere model of steric clashes between atoms, whereas potential energy may be more accurately represented by continuous functions of interatomic separation distances); likewise, electrostatic complementarity also might be more accurately described, for example, in terms of differential energetic contributions due to charged and uncharged hydrogen-bonding partners. However, typical epitope binding actually may be suboptimal considering thermodynamic and kinetic constraints on affinity maturation in B-cell ontogeny and the typical absence of affinity maturation in T-cell ontogeny, which may underlie heteroclitic cross-reaction wherein antigen is bound with higher affinity than immunogen.

Notwithstanding the problem of suboptimal complementarity described above, data presented in Table 2 suggest that the approach to epitope sequence redundancy proposed herein is more conceptually and practically meaningful than setting threshold (i.e., cutoff) values for fractional aligned-sequence identity. This is exemplified by the control-reaction epitopes of data rows 18 and 19 (having consensus sequence LLXRDSFEV). These epitopes differ from each other at just one residue position. As typical MHC class I-restricted T-cell epitopes, they are nonapeptides, for which the highest meaningful sequence-identity threshold value is 8/9 (~89%). For longer epitopes (e.g., typical MHC class II-restricted T-cell epitopes), the value is even higher. For example, in the case of data rows 4 and 5 (having consensus sequence NTWTTCQSIAXPSK), the said value is 13/14 (~93%). The threshold values thus calculated are markedly higher than those used (e.g., 70% [20]) for conventional protein sequence analysis. Moreover, regardless of the exact sequence lengths, applying such threshold values would entail discarding data on epitopes differing by one residue (e.g., the examples just cited from Table 2). To avoid such loss of potentially valuable information, all the available epitope data might be analyzed and a corresponding reduced epitope count reported to indicate the level of redundancy in the underlying dataset.

### 3.3. Survey of Peptidic Epitope Sequence Data

**Figure 6:**
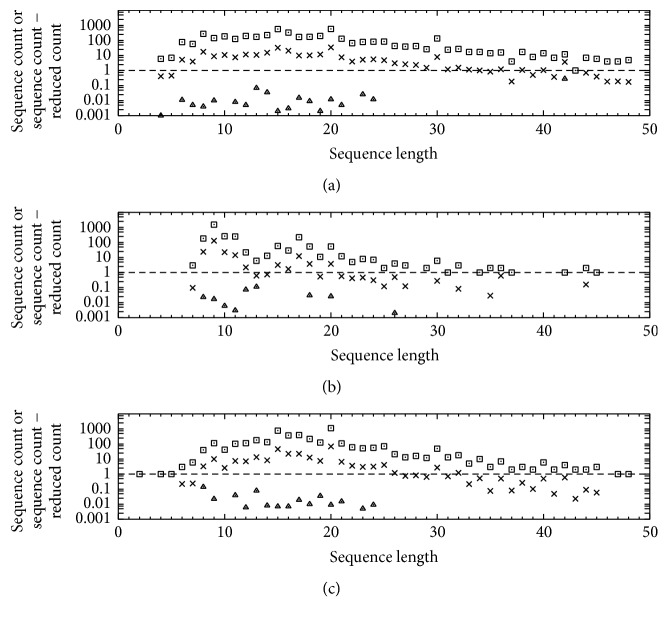
Peptidic epitope sequence data from IEDB for B-cell assays (a) and for T-cell assays of MHC restriction class I (b) or II (c). Sequence counts (“□”) are total epitope counts in IEDB. Corresponding reduced counts (*r* in (1)) are expressed as the difference (sequence count − reduced count), with functional similarity equated with either fractional aligned-sequence identity (“×”; (3) and (4)) or the Shannon information entropy for differential epitope binding (“△”; (5) through (9)). Missing “□” indicates sequence count = 0 (e.g., for B-cell epitope sequence length < 4 in (a)), where “□” is present and missing “×” or “△” indicates sequence count − reduced count = 0 (e.g., with missing “△” for B-cell epitope sequence length = 5 in (a)). Both “×” and “△” are missing where sequence count = 1 (e.g., for B-cell epitope sequence length = 43 in (a)), as reduced count = 1 for sequence count = 1.

IEDB searches yielded 11497 records on peptidic epitopes of length less than 50 residues (i.e., the general cutoff provided in the IEDB curation manual [39]), curated as covalently unmodified and comprising 4443 B-cell and 7054 T-cell epitope records (the latter divided between 2749 and 4305 records for MHC restriction classes I and II, resp.), for which computed functional redundancy vis-a-vis sequence length is depicted in Figure 6.

Considering the wide range of sequence counts for the various sequence lengths in Figure 6, a logarithmic scale was chosen to depict the data in a compact form. Consequently, the difference calculated as sequence count − reduced count was plotted instead of reduced count itself where sequence count > reduced count, to avoid crowding of data points. For all positive sequence counts (“□”), the said differences were consistently higher where functional similarity was equated with fractional aligned-sequence identity (“×”) rather than the Shannon information entropy of the differential epitope binding (“△”). In the latter case (“△”), the differences were consistently less than 1, with reduced count close or even equal to sequence count. These results are anticipated in view of Figure 4, which shows that reduced count is independent of sequence length using the entropy-based approach proposed herein but decreases with sequence length using fractional aligned-sequence identity.

The data in Figures 4 and 6 thus point to key considerations in evaluating functional redundancy. Clearly, functional redundancy may be overestimated by simply equating functional similarity with fractional aligned-sequence identity, especially with increasing sequence length for highly similar sequences. On the other hand, functional redundancy may be underestimated where functional similarity is equated with the Shannon information entropy of differential epitope binding if the sequence under consideration extends beyond the relevant epitope structure, which becomes more likely with increasing sequence length. Hence, the information-entropy approach as developed herein is conceivably applicable to sequences not exceeding a realistic application-dependent epitope length (e.g., six residues for antigen binding by anti-peptide antibodies [40]), although rigorous generalization to longer sequences might be pursued by aligning subsequences of such length and possibly accounting for differential immunodominance among immunogen epitopes [41]. For example, if a set of peptides were to be found such that each of them comprised a single immunodominant epitope having fixed length (e.g., six residues in all cases), the information-entropy approach might be applied to the entire set even if the peptides were of variable length. Moreover, even if the said immunodominant epitopes were highly similar to one another in terms of fractional aligned-sequence identity, all the available data still could be included in subsequent analyses, provided that both the total and reduced epitope counts would be reported in order to account for functional redundancy. In this way, useful epitope data could be retained instead of discarded.

### 3.4. Applications and Future Directions

The discussion thus far suggests the potentially greater utility of antigenic functional similarity cast as the Shannon information entropy of differential epitope binding (instead of fractional sequence-alignment identity) as basis for computing functional redundancy of epitope data to express their structural diversity in a biologically meaningful manner (i.e., explicitly in terms of relative binding affinity, which underlies the balance between the inversely related emergent continuum phenomena of immunologic specificity and cross-reactivity). This is subject to the caveat that assuming an idealized epitope-binding site of optimal steric and electrostatic complementarity (e.g., comparable to what is typically observed in a natively folded wild-type protein) may both overestimate affinity for an immunogen epitope and underestimate affinity for antigen epitopes structurally different from the immunogen epitope (thereby possibly overestimating immunologic specificity as a consequence of underestimating potential for cross-reactivity). These considerations could guide the development of immunization strategies (e.g., via vaccination) and immunodiagnostics (e.g., to produce peptidic constructs suitable for eliciting anti-peptide antibodies that either protect against disease or serve to detect particular antigens in biological samples via immunoassays), noting that the practical significance of immunologic specificity and cross-reactivity is highly context-dependent.

From the perspective of immunization, immunity (e.g., induced via vaccination or passive transfer of immune-system components) may be useful if specifically directed against a pathogen epitope so as to favor pathogen elimination without producing harmful hypersensitivity reactions (e.g., in the form of allergy or autoimmunity). Yet, such immunity might be even more useful if it also favored elimination of multiple antigenically related variant pathogens via cross-reaction (such that a single vaccine epitope elicited the production of immune-system components each cross-reactive with multiple variant pathogen epitopes) while still avoiding hypersensitivity. Cross-reactivity of immune responses is thus potentially useful within limits defined by risk of harmful hypersensitivity (e.g., due to cross-reaction with host self-antigens). As regards immunodiagnosis, an immunodiagnostic test may be useful if it enables specific pathogen detection via discrimination between a pathogen epitope and a set of other biologically relevant epitopes (e.g., of the host or its commensal symbionts in health, or of other pathogens for which the clinical implications are very different). Yet, the test might be even more useful if it also enabled detection of multiple antigenically related variant pathogens (such that a single immunologic probe detected multiple variant pathogen epitopes) while still retaining its discriminatory power with respect to other biologically relevant epitopes, particularly where the variant pathogens are very similar to one another in terms of their clinical implications (e.g., prognosis and therapeutic options). Hence, cross-reactivity strongly influences the outcomes of both immunization and immunodiagnosis, for which reason their development could be supported by computational tools that estimate epitope-binding affinity for cross-reactions.

In order to estimate the epitope-binding affinity of an immune-system component (e.g., antibody) elicited in response to (and thus having a binding site for) immunogen epitope *i* for antigen epitope *j*, one possible approach would be to express affinity in terms of association constants, for example, as

(10) $$\begin{array}{c} K_{ij}=K_{ii}Z_{ij}, \end{array}$$

where *K*_*ij*_ is the association constant for cross-reaction with antigen epitope *j*, *K*_*ii*_ is the association constant for reaction with immunogen epitope *i*, and *Z*_*ij*_ is a correction term to account for the difference between the two association constants. In turn, *K*_*ii*_ might be estimated as

(11) $$\begin{array}{c} K_{ii}=\exp\left(\;-\frac{\Delta G_{ii}}{RT}\;\right), \end{array}$$

where Δ*G*_*ii*_ is the free-energy change for reaction with immunogen epitope *i* while both *R* and *T* retain their meanings as in (7). In cases where epitopes *i* and *j* are both *m* residues in length, *Z*_*ij*_ might be estimated as

(12) $$\begin{array}{c} Z_{ij}=\left\{\begin{array}{cc} \overset{m}{\underset{k=1}{\prod }}s_{ijk}, & \text{if }\overset{m}{\underset{k=1}{\prod }}s_{ijk}>K_{ii}^{-1}\\ K_{ii}^{-1}, & \text{otherwise}, \end{array}\right. \end{array}$$

where the product retains its meaning as in (6), such that *K*_*ij*_ ≥ 1 (with *K*_*ij*_ = 1 for nonspecific binding). Δ*G*_*ii*_ could be estimated from epitope structure (e.g., using structural energetics [40–42]) subject to mechanism-specific constraints (e.g., on B-cell affinity maturation [43]); and *s*_*ijk*_ could be estimated along similar lines with regard to ΔΔ*G*_*ijk*_ in (7).

The preceding discussion has alluded to epitope binding as if it were a binary interaction, which is strictly true only for B-cell epitopes (e.g., bound by surface immunoglobulin or antibody); but in the context of T-cell epitopes, the epitope-binding site is to be understood as a bipartite entity consisting of an MHC molecule and a T-cell receptor (TCR), such that the epitope becomes at least partly confined between the two binding-site components. The overall process of T-cell recognition is subject to thermodynamic and kinetic constraints on initial MHC-peptide binding [44] and subsequent TCR recognition of MHC-peptide complex [45], which potentially complicate prediction of T-cell cross-reactivity and functional consequences thereof (e.g., considering how binding affinity and receptor numbers interact to influence T-cell effector function [45]). Still, predicting B-cell epitope cross-reactivity is challenging in view of the greater structural diversity of B-cell epitopes, especially where only a fraction of the epitope surface contributes to the epitope-paratope interface, such that cross-reaction may occur with variations in epitope structure beyond the interface. Hence, although the analysis herein for continuous epitopes might be generalized to discontinuous epitopes with indexing of residues by relative sequence positions (instead of consecutive sequence-position numbers) that accounts for any sequence-alignment gaps, correlating variations in epitope-residue structure with potential for cross-reactivity would be nontrivial.

The analysis developed herein conceivably could be refined to better account for MHC-peptide binding as distinct from subsequent binding of MHC-peptide complex by TCR, cognizant of MHC polymorphism. Hence, MHC-peptide binding could be analyzed with explicit consideration of pertinent MHC structural details (e.g., noting that unfavorable peptide backbone conformations may preclude accommodation of peptide within the MHC binding cleft and emphasizing the potential affinity contributions of prospective anchor residues on peptides vis-a-vis corresponding MHC binding pockets). Subsequent binding of MHC-peptide complex by TCR could be analyzed separately, with emphasis on peptide surfaces accessible for contact by TCR (e.g., excluding those buried within MHC binding pockets). This could be more readily applied to class I rather than class II MHC molecules, as the latter typically have binding clefts that allow for variable-length peptide overhangs. The analysis for MHC-peptide binding might be extended for class II via docking simulations to predict the MHC-bound conformations and affinity contributions of the overhangs, after which the binding of MHC-peptide complex by TCR could be analyzed with inclusion of any overhang surfaces that are likely to be accessible for contact by TCR.

At a more fundamental level, apparently suboptimal complementarity (manifesting as submaximal immunologic specificity) may be an inherent tradeoff in attaining optimal immune function from the standpoint of biological fitness (e.g., enabling each immune-system component to recognize a variety of structurally distinct epitopes, albeit at the cost of binding each of these epitopes with only submaximal affinity). Investigation of this possibility may lead to insights on how immune responses might be optimally biased (e.g., via vaccination or immunotherapy). Moreover, the asymmetry of cross-reaction with respect to immunogen- and antigen-epitope structures cautions against conceptualizing antigenic differences in terms of antigenic distance as a metric that is independent of immunization history.

## 4. Summary and Conclusions

In assembling epitope datasets (e.g., to develop and benchmark epitope-prediction tools), sequence redundancy among epitopes must be considered to avoid misleading results that reflect overrepresentation of functionally similar epitopes. However, potentially useful data may be needlessly discarded by excluding epitopes that share an apparently high degree of sequence similarity, as even only a single-residue difference may manifest as extreme functional dissimilarity between epitopes. The present work thus introduces a reduced epitope count, as defined using (1) and (2), to account for functional redundancy of epitope data (FRED) within an epitope dataset (such that FRED is quantified as the difference between the total and reduced epitope counts). This can be used, for example, to characterize the dataset instead of excluding epitopes with apparently similar sequences from it, thereby maximizing the use of available epitope data.

More importantly, the present work frames FRED in terms of potential antigenic cross-reactivity (rather than sequence distance) construed as functional similarity between epitopes. Hence, pairwise comparison of epitopes is performed such that each epitope pair considered is regarded as consisting of an immunogen epitope (which elicits the immune response of interest) and an antigen epitope (which is the target of cross-reactive binding in an immunoassay to evaluate the immune response). Functional similarity between epitopes is thus defined in a physicochemically and biologically meaningful way as the Shannon information entropy for differential epitope binding in (5), which is compatible with the possibility of extreme functional dissimilarity between epitopes due to seemingly minor differences in sequence. Consequently, the complement of functional similarity is not a distance (in the sense of a unique value quantifying the separation between two points in sequence space), as its value may vary with reversal of the roles (i.e., immunogen or antigen) assigned to epitopes under pairwise comparison. However, it is nonetheless a measure of epitope dissimilarity, such that summation of its values over all epitopes of a dataset according to (2) enables calculation of an averaged contribution of each epitope to the reduced epitope count. This circumvents the conventional dichotomous labeling of epitopes as either redundant or nonredundant based on an arbitrarily selected threshold value of sequence similarity. Such labeling is problematic in that selection of the actual threshold value is difficult to justify on physicochemically and biologically meaningful grounds and because an epitope thus might be labeled as redundant on the basis of similarity to a minority of other epitopes in the dataset.

In line with the preceding considerations, epitope functional similarity may be estimated in terms of differences between immunogen- and antigen-epitope structure relative to an idealized binding site of high complementarity to the immunogen epitope. However, this tends to underestimate potential for cross-reactivity, which suggests that epitope-binding site complementarity is typically suboptimal. The apparently suboptimal complementarity may reflect a tradeoff to attain optimal immune function that favors generation of immune-system components each having potential for cross-reactivity with a variety of epitopes. Such cross-reactivity conceivably could be exploited in the development of practical applications such as vaccines and immunodiagnostics (e.g., to aim for beneficially broad cross-reactivity rather than overly extreme immunologic specificity).

## Figures and Tables

**Table 1:** Numbers of unsatisfied hydrogen bonds for amino-acid residue substitutions of Figure 2, calculated according to (9).

	G	A	S	C	D	T	P	N	V	E	Q	H	L	I	M	K	R	F	Y	W
G	0	—	—	—	—	—	—	—	—	—	—	—	—	—	—	—	—	—	—	—
A	0	0	—	—	—	—	—	—	—	—	—	—	—	—	—	—	—	—	—	—
S	1	1	0	—	—	—	—	—	—	—	—	—	—	—	—	—	—	—	—	—
C	0	0	1	0	—	—	—	—	—	—	—	—	—	—	—	—	—	—	—	—
D	2	2	—	—	0	—	—	—	—	—	—	—	—	—	—	—	—	—	—	—
T	1	1	0	1	—	0	—	—	—	—	—	—	—	—	—	—	—	—	—	—
P	1	1	—	—	—	—	0	—	—	—	—	—	—	—	—	—	—	—	—	—
N	2	2	—	—	2	—	—	0	—	—	—	—	—	—	—	—	—	—	—	—
V	0	0	1	0	—	1	—	—	0	—	—	—	—	—	—	—	—	—	—	—
E	2	2	3	2	—	—	—	—	—	0	—	—	—	—	—	—	—	—	—	—
Q	2	2	3	2	—	—	—	—	—	2	0	—	—	—	—	—	—	—	—	—
H	2	2	—	—	—	—	—	—	—	—	—	0	—	—	—	—	—	—	—	—
L	0	0	1	0	—	—	—	—	—	—	—	—	0	—	—	—	—	—	—	—
I	0	0	1	0	—	1	—	—	0	—	—	—	—	0	—	—	—	—	—	—
M	0	0	1	0	—	—	—	—	—	—	—	—	—	—	0	—	—	—	—	—
K	1	1	2	1	—	—	—	—	—	—	—	—	—	—	1	0	—	—	—	—
R	3	3	4	3	—	—	—	—	—	—	—	—	—	—	3	4	0	—	—	—
F	0	0	—	—	2	—	—	2	—	—	—	—	—	—	—	—	—	0	—	—
Y	1	1	—	—	3	—	—	3	—	—	—	—	—	—	—	—	—	1	0	—
W	1	1	—	—	—	—	—	—	—	—	—	1	—	—	—	—	—	—	—	0

**Table 2:** IEDB Qualitative-Outcome (Q) data on peptide cross-reactivity assays (cf. Figure 5).

#	Q	Assay information				IEDB Epitope ID	Substitution	Sequence context (with *X* at position of substitution)	Ref.
		Type/MHC class	Parameter measured (cf. IEDB “measurement of” data field)	IEDB ID					
				Control reaction	Cross-reaction				
1	−	B-cell	Antibody-antigen binding	1370146	1370150	10801	W/Y	DXEDRYYRE	[22]
2	−	T-cell/I	Cytokine release (IFN-*γ*)	1340848	1340854	62604	P/A	SYIXSAEKI	[23]
3	−	T-cell/II	Cell proliferation	1646734	1646735	107384	E/D	GEPGIAGFKGXQGPK	[24]
4	−	T-cell/II	Cell proliferation	1660246	1660247	46317	F/A	NTWTTCQSIAXPSK	[25]
5	−	T-cell/II	Cytokine release (IL-4)	1660252	1660258	112245	A/F	NTWTTCQSIAXPSK	[25]
6	−	T-cell/II	Cytokine release (IL-2)	1667060	1667065	68846	K/A	VHFFXNIVTPRTP	[26]
7	−	T-cell/II	Cell proliferation	1667112	1667113	80071	A/K	VHFFXNIVTPRTP	[26]
8	−	T-cell/II	Cell proliferation	1716604	1716614	125569	T/V	SWEGVGVXPDV	[27]
9	−	T-cell/II	Cell proliferation	1716604	1716616	125569	D/N	SWEGVGVTPXV	[27]
10	−	T-cell/II	Cell proliferation	1716605	1716618	125571	V/T	SWEGVGVXPNV	[27]
11	+	T-cell/I	Cytotoxicity	1634784	1634785	103300	S/A	IYXTVAGSL	[28]
12	+	T-cell/I	Cytotoxicity	1634784	1634786	103300	G/S	IYSTVAXSL	[28]
13	+	T-cell/I	Cytotoxicity	1634788	1634789	103298	S/G	IYATVAXSL	[28]
14	+	T-cell/I	Cytotoxicity	1634788	1634790	103298	A/S	IYXTVASSL	[28]
15	+	T-cell/I	Cytotoxicity	1657362	1657364	111964	I/D	YXFAFRDL	[29]
16	+	T-cell/I	Cytokine release (IFN-*γ*)	1716442	1716443	125101	I/T	XMIKFNRL	[30]
17	+	T-cell/I	Cytokine release (IFN-*γ*)	1340848	1340852	62604	K/D	SYIPSAEXI	[23]
18	+	T-cell/I	Cytokine release (IFN-*γ*)	1381750	1381748	37174	D/G	LLXRDSFEV	[31]
19	+	T-cell/I	Cytokine release (IFN-*γ*)	1381752	1381753	37392	H/G	LLXRDSFEV	[31]
20	+	T-cell/I	Cytotoxicity	1404705	1404714	61086	S/I	SXIEFARL	[32]
21	+	T-cell/I	Cytotoxicity	1404705	1404715	61086	A/E	SSIEFXRL	[32]
22	+	T-cell/I	Cytotoxicity	1404705	1404716	61086	R/K	SSIEFAXL	[32]
23	+	T-cell/I	Cytotoxicity	1404712	1404719	61088	E/A	SSIEFXRL	[32]
24	+	T-cell/I	Cytotoxicity	1404713	1404721	61085	K/R	SSIEFAXL	[32]
25	+	T-cell/I	Cytokine release (IFN-*γ*)	1865529	1865530	98995	T/F	SAPDXRPA	[33]
26	+	T-cell/I	Cytokine release (IFN-*γ*)	1865529	1865532	98995	A/L	SAPDTRPX	[33]
